# “Getting pregnant at a young age doesn’t mean your life’s going to end”: A qualitative inquiry with older AI/AN youth participants of an evidence-based teen pregnancy prevention program

**DOI:** 10.1371/journal.pgph.0005696

**Published:** 2026-06-01

**Authors:** Gabrielle S. Evans-Mitchell, Abagail Edwards, Barbara Harvey, Christopher G. Kemp, Abagail Walsh, Jennifer Richards

**Affiliations:** Department of International Health, Center for Indigenous Health, Johns Hopkins University,‌‌ Baltimore, ‌‌Maryland, United States of America; PLOS: Public Library of Science, UNITED STATES OF AMERICA

## Abstract

AI/AN youth experience inequities in sexual health outcomes, including higher rates of sexually transmitted infections (STIs) and teen pregnancies, that are compounded by barriers to healthcare access and a lack of culturally competent services. Navajo Nation youth follow the same trends of overall AI/AN youth with high rates of STIs, teen pregnancies, and behaviors that predict such outcomes, such as early sexual initiation and early substance use initiation. These alarming reproductive health inequities call for urgent and culturally tailored sexual health education for Navajo and AI/AN youth. *Respecting the Circle of Life* (RCL) is one of the first sexual and reproductive health programs to be developed and rigorously evaluated with AI/AN youth. Previous evaluations of RCL on the Navajo Nation found the program to be most effective with younger youth, aged 11–14. To better understand how the program can serve older youth, we investigated the experiences of youth aged 15–19 on the Navajo Nation who had previously participated in the RCL program as part of a demonstration study during and after the COVID-19 pandemic. A purposive sample of AI/AN youth participants (n = 6) was interviewed to solicit feedback and experiences from the program. Our thematic analysis revealed four key themes that reflect the experiences of older youth (ages 15 – 19) who previously participated in the RCL program: (1) interactive and group-based learning, (2) peer and facilitator dynamics, (3) balanced and culturally relevant content, and (4) shifts in knowledge and attitudes. Youth reported meaningful shifts in knowledge, confidence, and perspective regarding their sexual health. They also expressed a clear need for deeper cultural alignment and age-relevant content. As Indigenous communities continue to reclaim and revitalize their approaches to sexual and reproductive health, programs like RCL must evolve to reflect the realities, resilience, and aspirations of all youth.

## Introduction

American Indian/Alaska Native (AI/AN) communities face unique challenges related to sexual and reproductive health that are deeply rooted in a complex history of colonization, cultural disruption, and systemic neglect. For centuries, the traditional knowledge and practices of AI/AN peoples regarding sexuality, reproduction, and relationships were marginalized or erased [1–4]. Today, AI/AN youth experience inequities in sexual health outcomes, including high rates of sexually transmitted infections (STIs) and teen pregnancies, that are compounded by barriers to healthcare access and a lack of culturally competent services. When compared to their white counterparts, chlamydia and gonorrhea rates among AI/AN populations are 3.7 and 4.6 times higher, respectively [5]. In 2020, the birth rate among AI/AN youth (age 15 – 19) was 25.7 births per 1,000 females, making it the highest among all racial/ethnic groups [6].

With approximately 400,000 tribal members, the Navajo Nation is the largest of the 574 federally recognized tribes in the U.S. The Navajo Nation reservation spans 24,000 square miles across primarily Arizona, as well as parts of New Mexico and Utah. Navajo Nation youth follow the same trends of overall AI/AN youth with high rates of STIs, teen pregnancies, and behaviors that predict such outcomes, such as early sexual initiation and early substance use initiation. Although national teen pregnancy rates have been declining since 2010, the 2020 rate for Arizona AI/AN is still 2.5 times higher than that of non-Hispanic White youth [7]. This trend also holds for Arizona’s predominantly Navajo Nation counties, where the STI rates are in stark contrast to Arizona’s overall STI rates. For chlamydia and gonorrhea, the rates in predominantly Navajo counties are approximately 1.3 times the rates of Arizona chlamydia and gonorrhea [8]. The most alarming STI inequities are apparent with new and congenital syphilis. The rates of new and congenital syphilis in one Navajo Nation County in Arizona are 4.5 and 4.4 times the rate of Arizona’s overall new and congenital syphilis rates, respectively [8]. Congenital syphilis often results in infant mortality [9]. These alarming reproductive health inequities call for urgent and culturally tailored sexual health education for Navajo and AI/AN youth.

AI/AN communities have shown resilience by reshaping the conversation around sexual health by incorporating both traditional knowledge and modern healthcare approaches. Using the Protection Motivation Theory [10,11], a community- and evidence-based STI, HIV, and pregnancy prevention program, called Respecting the Circle of Life: Mind, Body, and Spirit (RCL), was designed in partnership with the Johns Hopkins Center for Indigenous Health for AI/AN youth and their trusted caregivers [12]. RCL consists of 9 lessons covering decision making, values, goal setting, communication, and knowledge about risk behaviors associated with HIV, other STDs, teen pregnancy, violence, and alcohol and drug use. Peer-group participants meet for 8 sessions that each last 120 minutes. There is also a single 2-hour session conducted with each youth and a parent or other trusted adult after the peer-group sessions. RCL is one of the first sexual and reproductive health programs to be developed and rigorously evaluated with AI/AN youth [13]. Previous evaluations of RCL on the Navajo Nation found the program to be most effective with younger youth, aged 11–14 [14]. Specifically, RCL was found to be effective in strengthening protective factors related to adolescent risk behaviors, noting improved attitudes around peer pressure to try drugs and increased youths’ willingness to talk with parents/caregivers about drugs and alcohol [14]. However, older AI/AN youth (ages 15 – 19) experience disproportionately higher rates of STIs and teen pregnancies compared to younger peers, yet limited research has examined how they specifically engage with or benefit from programs like RCL. Previous evaluations of RCL have largely focused on younger youth, leaving a gap in understanding its relevance in older youth. To address this gap, we investigated the experiences of youth aged 15–19 on the Navajo Nation who had previously participated in the RCL program as part of a demonstration study during and after the COVID-19 pandemic.

## Materials and methods

### Ethics statement

Ethical approval for this study was obtained from the Johns Hopkins Bloomberg School of Public Health Institutional Review Board (IRB18285) and the Navajo Nation Human Research Review Board (NNR-19.364). Informed oral consent was obtained from all participants.

The Navajo Nation RCL program was implemented by Indigenous staff at the Johns Hopkins Center for Indigenous Health from 2020 to 2022, serving youth aged 10–19. Originally designed to be delivered in a camp format starting the summer of 2020, the program delivery shifted to a virtual setting due to the COVID-19 pandemic [15]. During this time, virtual implementation of RCL was found to be feasible and generally acceptable; however, the shift away from in-person delivery appeared to constrain interaction and engagement, potentially limiting the program’s overall effectiveness. The original RCL program consists of nine lessons – eight lessons with groups of youth and one lesson delivered to an individual youth and trusted adult dyad in the home. The program was modified for virtual implementation to consist of 12 lessons, each shorter in length, with 10 lessons delivered to youth and two lessons to the youth and trusted adult dyad at the end of the program. In 2021, RCL began implementing in a hybrid format, with both virtual and in-person lessons adhering to COVID-19 safety protocols. A total of 164 youth and their trusted adults were enrolled in the program. The program was self-paced and consisted of self-selected peer groups, often siblings or relatives who resided in the same household.

A purposive sample of AI/AN youth participants (n = 6) who participated in RCL were interviewed to solicit feedback and experiences from the program. Participants were eligible if they were over 18 years old, identified as American Indian/Alaska Native, and had previously participated in the RCL program when they were between 15 and 19 years old. We only recruited participants over 18 years old due to the feasibility of obtaining informed consent and that a significant portion of youth in the 15–19 age range during the program turned 18 by the time of interview recruitment. The sample was recruited via flyers and direct contact from study staff. Participants received a $50 incentive for completing the interview. Interviews were conducted until saturation was reached, which was only six participants. We decided that our small sample size of youth was adequate because we did not see strong variation across responses in the interviews. Further, we did not elect to interview trusted adults as the focus of this study was to evaluate the youth-only lessons of the program. Participants were between 18 and 20 years old, with five out of six participants identifying as female, and the majority (5/6) participated in-person. The study was reviewed and approved by the appropriate academic and tribal review boards for human subject research.

Qualitative interviews were semi-structured, consisting of open-ended questions related to the appropriateness and fit of the program. An interview guide was developed by the study team based on the research questions and target audience. Key topics included program delivery, acceptability of the program from the youth’s perspective, impact of the program on the youth’s life, age appropriateness, importance of RCL program topics, and feedback on specific lessons/activities of the program. Interviews were conducted and recorded over Zoom between January and April 2022 by research staff not involved in program implementation. Interviews lasted 17 – 35 minutes and were transcribed using online software. Transcripts were coded using an inductive approach to thematic analysis in Microsoft Word by three coders, ‌‌two of whom were Diné (Navajo) and one who was Haliwa-Saponi, positioning the analysis and results from an Indigenous lens. Inductive approaches enabled coders to develop new codes as they read the transcripts, encouraging open-mindedness and an accurate reflection of the‌‌ data [16]. Transcripts were independently coded by each coder. Coders then met regularly to compare their coding, discuss discrepancies, and collaboratively refine code definitions to ensure consistency. Differences in coding were resolved through discussion and consensus. After each transcript was coded, reviewed, and refined, the codes were grouped into themes based on common patterns. The study team further discussed and finalized themes, using episodic personas to illustrate the common experiences of older youth in the program.

## Results

### Thematic overview

Our analysis revealed four key themes that reflect the experiences of older youth (ages 15 – 19) who previously participated in the RCL program: (1) interactive and group-based learning, (2) peer and facilitator dynamics, (3) balanced and culturally relevant content, and (4) shifts in knowledge and attitudes. While these themes may also resonate with younger youth, they take on distinct meaning for older youth, given their developmental stage, social roles, and lived experiences navigating relationships, family expectations, and cultural identity. Additionally, to demonstrate each theme, we developed personas based on participants’ experiences to reflect the perspectives of youth and provide insight into their participation in the RCL program.

### Interactive and group-based learning

Many of the youth shared that their most meaningful learning experiences came from the in-person, interactive and group-based learning. Youth engaged most with hands-on activities and visual learning tools. When sessions were moved online due to COVID-19, youth shared that the virtual sessions made it easier to be distracted and harder to make connections with facilitators and their peers.

“ I like how it was delivered, because she used poster boards to explain things visually. And then she used PowerPoints and video, which was very helpful, too. And it was hands-on, too.” - Participant 1, Female

“I didn’t know how to do that [how to put on a condom], but now I know. Just learning different stuff about the male body and the female body. Those were very helpful tips.” - Participant 5, Male

### Peer and facilitator relationship

Older youth described feeling awkward discussing personal topics at first, but over time they found comfort and ease. Strong, trusting relationships with facilitators were essential for engagement, and participants were more likely to open up when they felt facilitators genuinely cared and were familiar faces. Several youth expressed a desire for male facilitators and greater male peer involvement to make the program feel more inclusive. Many also shared that they were comfortable talking about sexual health with peers, rather than adults, highlighting the importance of a peer education model.

“[A facilitator] came over to the chapter house where we were working for the summer, and I decided to sign up. We are basically related, so I was comfortable working with [them] on the program.” -Participant 3, Female

“I told my sister about it because I know that she didn’t know about it, and to this day, I still think about the program. I want to just get that information to my other family members that never heard about the program.” -Participant 4, Female

### Balanced and culturally relevant content

While youth appreciated how much they learned about reproductive health, several expressed the desire for the inclusion of cultural content, specifically rooted in Navajo traditions and beliefs. Additionally, older youth hope for a more neutral approach to discussing teen pregnancy. They suggested incorporating a storytelling component featuring former teen parents to help dispel myths, break down stereotypes, and make the topic feel less stigmatized.

“I don’t really like to promote that idea due to my sister getting pregnant at a young age. I felt a need to defend her in a way. Getting pregnant at young age doesn’t mean your life’s going to end. It doesn’t mean that you won’t have the same opportunities as individuals who didn’t get pregnant. It’s just going to be harder and more power to you, that you were able to continue and create these accomplishments while you had a kid, while you were pregnant.” -Participant 6, Female

### Shifts in knowledge and attitudes

The program did more than just fill an information gap; it strengthened youths’ ability to make informed and intentional decisions about their sexual health. Participants described learning not just how to use condoms or prevent sexually transmitted infections, but also why these practices matter for their futures. This deeper understanding translated into greater confidence in setting boundaries and delaying sexual activity. Others described becoming more cautious and deliberate in their approach to sex, relationships, and contraception or barrier method use. Reactions to the discussions on teen pregnancy were mixed – while some found it eye-opening, others suggested the topic could be reframed to present a more balanced perspective.

“The RCL program taught me to wait for the right person because the right person will like, feel your emotions, and won’t just be there because you’re pretty and wants to have sex. Waiting for the right person when it comes to a relationship.” - Participant 3, Female

“I think it changed. Like, it’s okay, but just as long as you practice it safely. Most importantly, abstinence would be the key.” - Participant 2, Female

### Personas

Personas were developed based on the collective perspectives of participants. It is essential to note that the names assigned to the personas are not associated with actual study participants, and the provided ages represent participants’ ages at the time of their interviews.

### Persona 1: Mitchell, Male, Age 18 – Interactive and group-based learning

Mitchell thrives in collaborative environments and was fortunate enough to attend the RCL program in person. He enjoyed working in small groups with his peers and participating in hands-on activities such as condom demonstrations. Mitchell also felt that the icebreakers helped warm everyone up and build connections, which made activities such as the condom demonstration less awkward. Although Mitchell thoroughly enjoyed the program, he found himself wishing there had been male facilitators and more male peers with whom he could connect and relate.

### Persona 2: Jordan, age 20 – Peer and Facilitator Dynamics

Jordan was unsure about participating in a sexual health program, but her parents thought it’d be a great learning opportunity. She gave it a try and started to engage more after feeling a connection with one of her facilitators. Jordan appreciated how the facilitator was fully present and patient with her while she warmed up to the program. Both Jordan and her peers were also more open to participating because they had a facilitator who looked like them. Despite being unsure about participating in the program, Jordan found herself sharing what she learned with her younger cousins – the information that she learned was very important. When she was younger, she wished she had an older sibling or cousin who would’ve shared this information with her.

### Persona 3: Gwen, age 18 – Balanced and Culturally Relevant Content

Gwen is proud of her American Indian heritage and often reflects on how her Diné identity and values intersect with her health. She found the curriculum informative and helpful but occasionally felt disconnected from it. She wished that the content was more culturally grounded and spoke directly to Navajo teachings on topics such as relationships/kinship and body knowledge. Additionally, she hoped for a more neutral approach to discussing teen pregnancy, as opposed to speaking negatively about it. She also believed that it may be impactful to hear from former AI teen parents on their experiences.

### Persona 4: Alex, age 20 – Shift in Knowledge and Attitudes

Like many of their peers, Alex received limited sexual health education in school and wasn’t too sure how accurate the information was that they received from social media and TV/movies. When they attended RCL, they were both curious about the new information they’d be receiving, but also unsure about how comfortable they’d feel. Over time, Alex became more confident in their knowledge of STIs, contraception options, and overall sexual health. They were also able to learn what information was truly accurate and move past any myths that they’d once believed to be true. RCL not only helped them increase their awareness of sexual health risks and prevention methods but also shift to a more reflective attitude towards relationships, sexual choices, and informed decision-making.

## Discussion

This paper analyzes accounts from six older participants who previously participated in the RCL program on the Navajo Nation. Thematic analysis revealed four key insights: the value of interactive and group-based learning, the importance of peer and facilitator dynamics, a call for more balanced and culturally relevant content, and positive shifts in knowledge and attitudes regarding youth’s own sexual health behaviors. Participants emphasized the need for hands-on activities, trusted facilitators, and content that reflects lived experiences. While these themes may resonate across age groups, how older youth interpret and apply their experiences reflects developmental priorities unique to late adolescence, including a desire for more nuanced discussions of teen pregnancy, greater readiness for future planning and boundary-setting, and an emerging sense of responsibility to share knowledge with younger peers. Although participant feedback varied in how they internalized and applied what they learned, all reported gaining critical information that influenced how they view relationships, sexual health, and their futures.

These findings are broadly consistent with previous evaluations of the RCL program, particularly those demonstrating its effectiveness with younger AI/AN youth [14]. However, this study contributes to the literature by highlighting the distinct needs and perspectives of older youth, a group that has shown weaker behavioral outcomes in prior RCL evaluations. Specifically, older participants desired more nuanced discussions of pregnancy, more visible male engagement, and stronger integration of Indigenous perspectives. These findings align with the broader literature emphasizing the importance of Indigenous sexual health programming that integrates traditional knowledge, challenges, stigmas, and respects youth autonomy [3–5].

There are several strengths and limitations to consider. A key strength of the study is the cultural relevance and positionality of the research team, including program implementers from the local community and two Diné (Navajo) coders who contributed to the data analysis, yielding more grounded thematic insights. However, it is essential to acknowledge that this paper also has limitations. Although saturation was reached, delays and challenges with recruitment contributed to the small sample size of n = 6, which may not represent all perspectives and experiences. Another notable limitation is the absence of interviews with youth under 15 at the time of participation. This decision was made to focus on understanding how the RCL program can better serve older youth. However, this exclusion prevents us from comparing the experiences of younger participants with those of older youth, thereby limiting the generalizability of the findings. A second limitation is that social desirability bias is a likely factor, as, participants may have been more inclined to provide positive feedback rather than negative feedback. Lastly, the findings may not be generalizable beyond the context in which they were obtained (i.e., the Navajo Nation), as American Indian/Alaska Native cultures vary across tribes/nations and regions. Despite these limitations, this study offers valuable insights and several practical implications for how the RCL program and other comprehensive sexual education programs can better serve older youth.

Practical implications include highlighting the importance of developmental tailoring, specifically how older youth benefit from different program contexts and delivery styles than younger youth participants. Secondly, incorporating culturally grounded elements, such as traditional teachings and peer facilitation, may enhance program impact and resonance. Third, involving male facilitators and encouraging male youth participation may create a more inclusive environment. Lastly, the results underscore the value of peer-to-peer learning and community-driven adaptations in enhancing Indigenous knowledge, autonomy, and resilience. Future iterations of RCL should consider these adaptations to support older Native youth better as they navigate sexual health and identity in both culturally affirming and developmentally appropriate ways.

## Conclusion

Older AI/AN youth who participated in the RCL program reported meaningful shifts in knowledge, confidence, and perspective regarding their sexual health. They also expressed a clear need for deeper cultural alignment and age-relevant content. Findings from this study will inform adaptations to RCL that better meet the needs of older youth, including incorporating culturally grounded content rooted in Tribal values and traditions, integrating storytelling from Native teen parents to provide more balanced perspectives, and expanding opportunities for peer-led discussion and engagement. Additionally, adaptations may include increasing the representation of male facilitators and participants, refining content to reflect the developmental realities of older adolescents, and emphasizing interactive, ‌‌discussion-based learning.

As Indigenous communities continue to reclaim and revitalize their approaches to sexual and reproductive health, programs like RCL must evolve to reflect the realities, resilience, and aspirations of all youth. Culturally responsive, peer-informed, and age-tailored adaptations are not only warranted but essential.

## Supporting information

S1 Checklist COREQ Checklist. (PDF)

S1 Text Individual Interview Guide. (DOCX)
