# Supplementary material for: “Getting pregnant at a young age doesn’t mean your life’s going to end”: A qualitative inquiry with older AI/AN youth participants of an evidence-based teen pregnancy prevention program
Source: PLOS Glob Public Health. 2026 Jun 1;6(6):e0005696. doi: 10.1371/journal.pgph.0005696 (PMC13225420; doi:10.1371/journal.pgph.0005696)
Supplement: S1 Text — (DOCX) [file pgph.0005696.s002.docx]

**RCL Older Youth Interview Guide**

The following questions will ask you about your experiences as a participant in the Respecting the Circle of Life (RCL) program. There are no right or wrong answers. Please answer honestly - your answers to these questions will help us to develop a better version of the program that is designed specifically for youth ages 15-19 years old. We will not use your name or any other identifying information when we look at responses to these questions. First, I will ask you to share your thoughts on the content of the RCL lessons. Next, I will ask you to share your thoughts on how the RCL program should be delivered.

*These first few questions will ask about RCL program content.*

1. **Tell me about your experience in the program**
   1. How did you do RCL (virtual or in person)?
   2. Tell me about your group:
      1. How many other youth were in your sessions with you?
      2. Were you able to schedule the lessons easily?
2. **What did you think of RCL?**
   1. What did you enjoy most about it?
   2. What did you not enjoy?
3. **Have you talked to your friends about RCL? Why or why not?**
4. **Did you think the RCL program was important for youth your age? Please explain why or why not.**
   1. What information was the most helpful? Why?
   2. What information was the least helpful? Why?
   3. Did you learn new information? What did you learn that you thought was most important to you?
5. **What kind of topics related to sexual or reproductive health are you most interested in learning about? (e.g. probe on pregnancy, STDs, puberty, relationships, etc.)**
   1. Did RCL provide information about this topic?
      1. If yes, was it enough?
6. **Are there other things or topics that you wish the RCL program would have covered?**
   1. If yes, what are they? What do you want to learn about these topics?
7. **Are there any lessons or activities that you feel could be taken out of the program?**
8. **What would you change about the program to make it more interesting for youth ages 15-19?**
9. **How did participating in RCL impact you?**
   1. Did your attitude about sex change? How?
   2. Did your attitude about condoms and contraception change? How?
   3. Did any of your relationships change?
      1. With your partner (boyfriend/girlfriend)? How?
      2. With your friends? How?
      3. With your parents or caregiver? How?
      4. With anyone else? How?
   4. Is there anything else that has changed in your life because of the program?

*These next few questions will ask about RCL program delivery.*

1. **Overall, did you like the way the RCL program was delivered? Please explain why or why not.**
   1. Did you like the delivery method (virtual or in person)?
   2. Did you like who it was delivered to (one-on-one or group format)?
   3. Would you like to do this program on your own, without someone delivering it to you, like on your phone or on a computer?
   4. What would you change about how RCL is delivered?

*My last question can be about any part of the RCL program (lessons, topics, activities, facilitators, technology, internet, etc.).*

1. **Do you have any ideas for how we can make the program better for youth ages 15-19?**

***Thank you for your feedback!***
